# Fungal Hybrid B heme peroxidases – unique fusions of a heme peroxidase domain with a carbohydrate-binding domain

**DOI:** 10.1038/s41598-017-09581-8

**Published:** 2017-08-24

**Authors:** Marcel Zámocký, Štefan Janeček, Christian Obinger

**Affiliations:** 1 Department of Chemistry, Division of Biochemistry, University of Natural Resources and Life Sciences (BOKU), A-1190 Vienna, Austria; 2 Institute of Molecular Biology, Slovak Academy of Sciences, SK-84551 Bratislava, Slovakia; 3 Department of Biology, Faculty of Natural Sciences, University of SS Cyril and Methodius, SK-91701 Trnava, Slovakia

## Abstract

Heme peroxidases, essential peroxide converting oxidoreductases are divided into four independently evolved superfamilies. Within the largest one – the peroxidase-catalase superfamily - two hybrid lineages were described recently. Whereas Hybrid A heme peroxidases represent intermediate enzymes between ascorbate peroxidases and cytochrome *c* peroxidases, Hybrid B heme peroxidases are unique fusion proteins comprised of a conserved N-terminal heme peroxidase domain and a C-terminal domain of various sugar binding motifs. So far these peculiar peroxidases are only found in the kingdom of Fungi. Here we present a phylogenetic reconstruction of the whole superfamily with focus on Hybrid B peroxidases. We analyse the domain assembly and putative structure and function of the newly discovered oligosaccharide binding domains. Two distinct carbohydrate binding modules (CBM21 and CBM34) are shown to occur in phytopathogenic ascomycetous orthologs of Hybrid B heme peroxidases only. Based on multiple sequence alignment and homology modeling the structure-function relationships are discussed with respect to physiological function. A concerted action of peroxide cleavage with specific cell-wall carbohydrate binding can support phytopathogens survival within the plant host.

## Introduction

Peroxidases (EC 1.11.1.1–1.11.1.19) are essential peroxide converting oxidoreductases present in all domains of life. Four heme peroxidase superfamilies (namely: peroxidase-catalase, peroxidase-cyclooxygenase, peroxidase-chlorite dismutase and peroxidase-peroxygenase) arose independently during evolution^1, 2^. They differ in overall fold, active site architecture and enzymatic activities, catalysing the hydrogen peroxide-mediated one- and two-electron oxidation of a myriad of cationic or anionic inorganic and organic molecules or even proteins (Reactions 1 and 2). Additionally, efficient dismutation of H_2_O_2_ can be performed by some representatives (Reaction 3).

1 $${{\rm{H}}}_{{\rm{2}}}{{\rm{O}}}_{{\rm{2}}}+2{{\rm{AH}}}_{{\rm{2}}}\to 2{{\rm{H}}}_{{\rm{2}}}{\rm{O}}+2{{\rm{HA}}}^{\bullet }$$

2 $${{\rm{H}}}_{{\rm{2}}}{{\rm{O}}}_{{\rm{2}}}+{\rm{HX}}\to {{\rm{H}}}_{{\rm{2}}}{\rm{O}}+{\rm{2HOX}}$$

3 $${{\rm{H}}}_{{\rm{2}}}{{\rm{O}}}_{{\rm{2}}}+{{\rm{H}}}_{{\rm{2}}}{{\rm{O}}}_{{\rm{2}}}\to 2{{\rm{H}}}_{{\rm{2}}}{\rm{O}}+{{\rm{O}}}_{{\rm{2}}}$$

The various physiological roles range from the degradation of hydrogen peroxide derived from aerobic life style or pathophysiological processes (Reactions 1 and 3) through H_2_O_2_-mediated formation of antimicrobial and halogenating oxidants (e.g. hypohalous acids, HOX, Reaction 2) to the production of radicals (HA^●^, Reaction 1). Peroxidase-formed radicals are involved in either polymerization reactions^3–5^, polymer modification^6^ or degradation reactions like plant cell wall degradation by white rot fungi^7^. The latter process recycles large amounts of carbon fixed by photosynthesis of land plants^8, 9^.

The peroxidase-catalase superfamily is the most abundant heme peroxidase superfamily currently counting over 8,800 unique annotated members in PeroxiBase^10^ (http://peroxibase.toulouse.inra.fr/) and many more putative sequences in general databases (Table 1). This superfamily was originally named plant, fungal, and bacterial peroxidase superfamily and primarily divided in three structural classes according to a typical, rather conserved fold of their main catalytic domain^11^. Since then, many attempts were made to analyse its phylogeny in detail^12–15^. In 2015 we suggested to divide the superfamily in three families (instead of classes) thus providing the same systematic nomenclature as used in other superfamilies^1^. Family I is comprised of (bifunctional) catalase-peroxidases, ascorbate peroxidases, cytochrome *c* peroxidases and all their evolutionary intermediates. In Family II fungal secretory peroxidases including all manganese and lignin peroxidases and their evolutionary intermediates like versatile peroxidases are found, but also numerous other peroxidases described yet as “generic” (expected to be nonlignolityc)^9^. Finally, Family III is represented by plant secretory peroxidases with hundreds of closely related genes in almost all sequenced genomes of the plant kingdom.

**Table 1:** Overview on all peroxidase and catalase families annotated in the InterPro database^17^ (http://www.ebi.ac.uk/interpro/) with numbers of matched proteins and count of various domain architectures (update June 2017).

Family #	Description new	Description old	Proteins matched	Architectures count
IPR024706	peroxiredoxins	AhpC type	37,193	13
IPR002016	peroxidase-catalase	“non-animal”	23,309	299
IPR018028	catalase typical	monofunctional	21,030	126
IPR000889	glutathione peroxidase	(the same)	17,317	72
IPR006314	peroxidase-dismutase	dyp-type	10,982	50
IPR019791	peroxidase-cyclooxygenase	“animal”	10,434	679
IPR004852	di-haem peroxidase	cyt. c peroxidase	8,522	104
IPR002065	thiol peroxidase	(the same)	7,367	12
IPR007760	manganese catalase	“pseudocatalase”	5,190	16
IPR000028	peroxidase-peroxygenase	chloroperoxidase	2,585	31

From an evolutionary point of view the phylogeny of the intermediates positioned between the three families is highly interesting. Important turning points of gene evolution are represented by (i) hybrid A or ascorbate-cytochrome *c* peroxidases^16^ and by (ii) Hybrid B heme peroxidases that were previously classified as Family I members^13^. However, recent analyses clearly demonstrated significant differences between Hybrid B peroxidases and Hybrid A or other Family I members^2, 14, 15^. In the present study we demonstrate that Hybrid B heme peroxidases are found solely in the kingdom of Fungi and are comprised of two domains, i.e. a conserved N-terminal catalytic peroxidase domain and a C-terminal carbohydrate-binding domain with a high variability. We present the phylogeny of these fungal enzymes, discuss their domain assembly and carbohydrate sequence motifs (CBMs) as well as their putative tertiary structures derived from homology modelling. Additionally, the physiological role of these oxidoreductases is discussed.

## Results and Discussion

### Phylogeny of the peroxidase-catalase superfamily

The peroxidase-catalase superfamily annotated in databases as IPR002016 or PF00141 is currently represented by more than 23,300 protein sequences. As Table 1 demonstrates it represents the largest superfamily of heme peroxidases in InterPro database^17^ and the second largest (super)family of hydrogen peroxide reducing heme enzymes (including monofunctional catalases). Because there were recent attempts to group quite different peroxidase and catalase sequences together in a cladogram (e.g. a neighbour-joining reconstruction^18^) it is important to note that the heme peroxidase superfamilies summarized in Table 1 arose during genome evolution independently from each other and from non-heme peroxidases and all catalases^2^ as explained also in PeroxiBase documentation at http://peroxibase.toulouse.inra.fr/infos/documentation.php.

The three families and twelve subfamilies (catalase-peroxidases, ascorbate peroxidases, ascorbate-peroxidase-related, ascorbate-peroxidase-like, cytochrome *c* peroxidases, manganese peroxidases, lignin peroxidases, versatile peroxidases, “generic” peroxidases, plant secretory peroxidases and hybrid peroxidases of type A & B) contain sequences from all domains of life. Definitively, there are now numerous novel members stemming from taxonomic lineages beyond bacteria, fungi and plants thus it is more appropriate to denominate the whole superfamily as peroxidase-catalase superfamily reflecting the dominance of peroxidase (Reactions 1 and 2) and catalase (Reaction 3) reactivities^1, 2^. The typical mainly α-helical fold of the catalytic domain including the architecture of the heme *b* cavity remained conserved during evolution of this superfamily. On the other hand, there is a rather high sequence variability in the heme periphery, around binding sites of electron donors^19^ and in non-essential regions.

**Figure 1:**
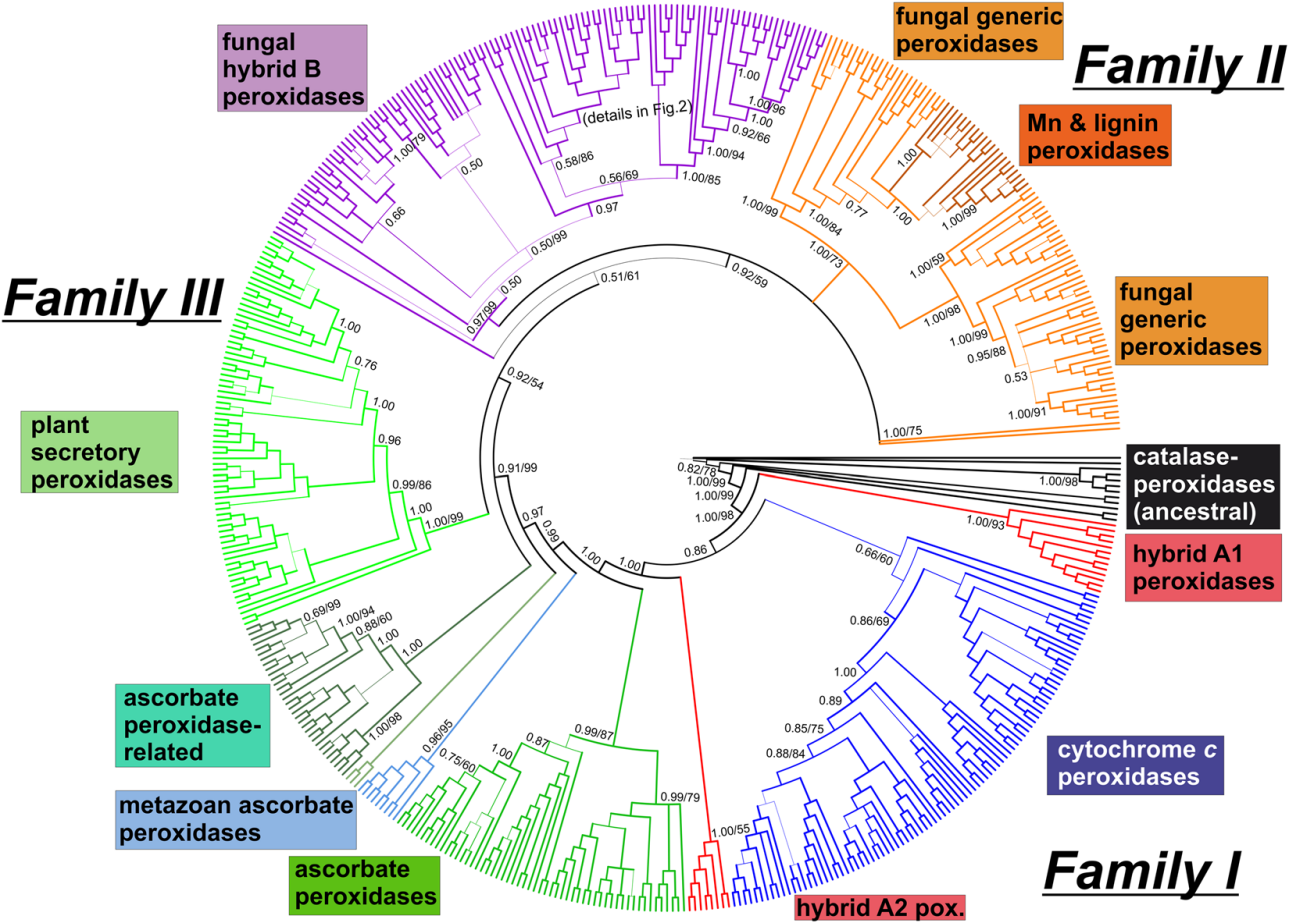
Global phylogenetic tree of the peroxidase-catalase superfamily. Presented is a circular tree for 500 full-length protein sequences obtained from Mr. Bayes (version 3.2) analysis with relative burn-in of 25%, WAG model of substitutions^20^, 4 gamma categories and sampling over 3,000,000 generations. A very similar tree was obtained also with the maximum likelihood method of the MEGA suite (version 7) with 100 boostrap replications and 4 gamma categories. Values in the (main) nodes represent posterior probabilities and bootstrap values (shown only values above 0.5/50), respectively. The thickness of the branches corresponds direct proportionally with obtained posterior probabilities. This evolutionary tree is deposited at http://itol.embl.de/.

Here we present a detailed molecular phylogeny of 500 full length protein sequences proportionally selected from all subfamilies mentioned above. For the phylogenetic reconstruction both MrBayes inference, version 3.2.6 with invariant gamma rates using the Whelan-Goldman model^20^ (Fig. 1), and Maximum Likelihood method based on the same Whelan-Goldman model implemented in MEGA 7 suite (Supplementary material 1) were used. It was already suggested^14^ that the ancient representatives of this superfamily were bifunctional catalase-peroxidases (Fig. 1). Catalytic promiscuity is often observed in ancient enzymes^21^. In the later course of evolution bifunctional catalase-peroxidases diverged stepwise into monofunctional peroxidases with distinct substrate specificities.

Family I is currently comprised of ancient catalase-peroxidases, cytochrome *c* peroxidases, hybrid A peroxidases (abbreviated as APx-CcP), which segregated in two different main clades, and various clades of ascorbate peroxidases. A recent study focused mainly on these divergent clades of ascorbate peroxidases^15^. Besides “classical” Family I ascorbate peroxidases and ascorbate-peroxidase related (APx-R) genes, which segregated in two well-supported clades (Fig. 1), a new subfamily named “ascorbate-peroxidase like” (APx-L) situated on the evolutionary way from Family I towards ancestors of Families III and II was suggested^15^. The question remains whether APx-R and APx-L can still use ascorbate as (main) electron donor. With respect to the peroxidase domain APx-Ls might also represent pseudogenes^15^. Our present phylogenetic analysis shows that Metazoan (clearly non-plant) putative ascorbate peroxidases descended from the basal clades directly after the branches of “classical” intracellular ascorbate peroxidases from red & green alga and plants (Fig. 1). Thus their common ancestor was already present during the formation of primordial eukaryotic cells. It is also evident that “ascorbate peroxidase-related” proteins were segregated in distinct clades probably sooner than Family III plant secretory peroxidases (Fig. 1).

Recently, we have described the occurrence of Hybrid B peroxidases and started to analyse their phylogeny^2, 14^. The present comprehensive phylogenetic reconstruction is mainly based on the Bayesian inference and a detailed comparative analysis of sequences. In contrast to Hybrid A peroxidases Hybrid B enzymes are strictly monophyletic (labelled violet in Fig. 1). With high statistical support the reconstruction reveals that there was a common ancestor for Family III enzymes (i.e. plant secretory peroxidases), Hybrid B peroxidases and all Family II descendants (i.e. manganese, lignin and all generic fungal secretory peroxidases) (Fig. 1). From a survey within PeroxiBase^10^ it can be expected that already the common ancestor of all known Hybrid B and Family II fungal peroxidases was a secretory protein. Genes for Hybrid B peroxidases can be found in the earliest diverging fungal lineage, in Chytridiomecetes (e.g. BdeHyBpox1 sequence from *Batrachochytrium dendrobatidis*). In contrast, there is no known sequence of a Family II representative found in a phylogenetically basal lineage of Fungi yet. Family II enzymes (generic, manganese & lignin peroxidases) occur in Dikarya (Ascomycetes & Basidiomycetes) only. Thus, Hybrid B peroxidases appear to have older roots than all Family II members but clearly more sequences from all basal fungal lineages are necessary to strengthen this hypothesis.

**Figure 2:**
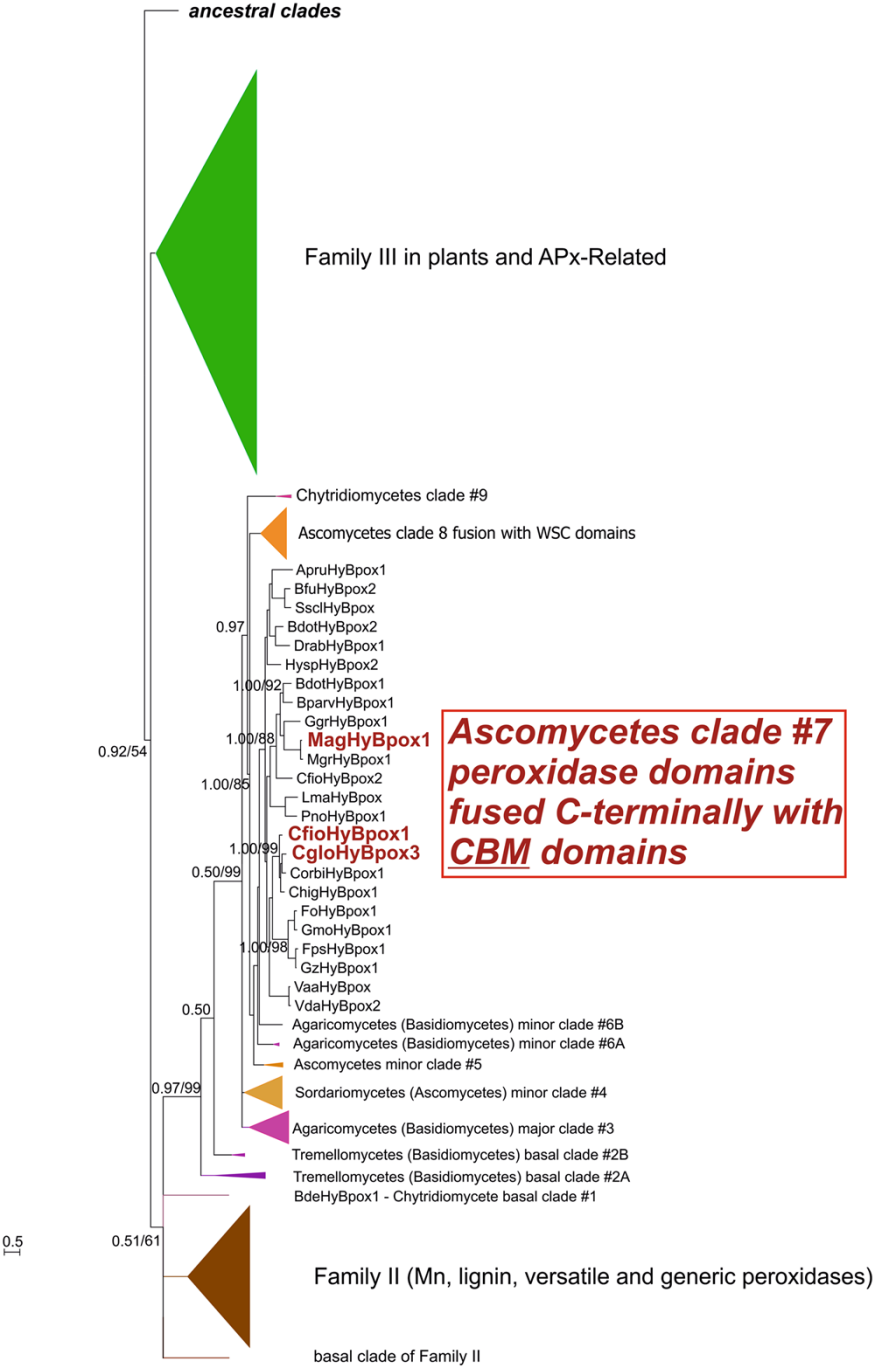
Detail of the evolutionary tree focused on Hybrid B peroxidases. Presented are those clades of the global peroxidase-catalase superfamily tree (Fig. 1) which show relationships among ascomycetous Hybrid B heme peroxidases possessing various carbohydrate binding domains. Values in the nodes represent posterior probability and bootstrap values (shown only above 0.5/50), respectively. Abbreviations of used peroxidase names are listed in Supplementary Table 1.

**Figure 3:**
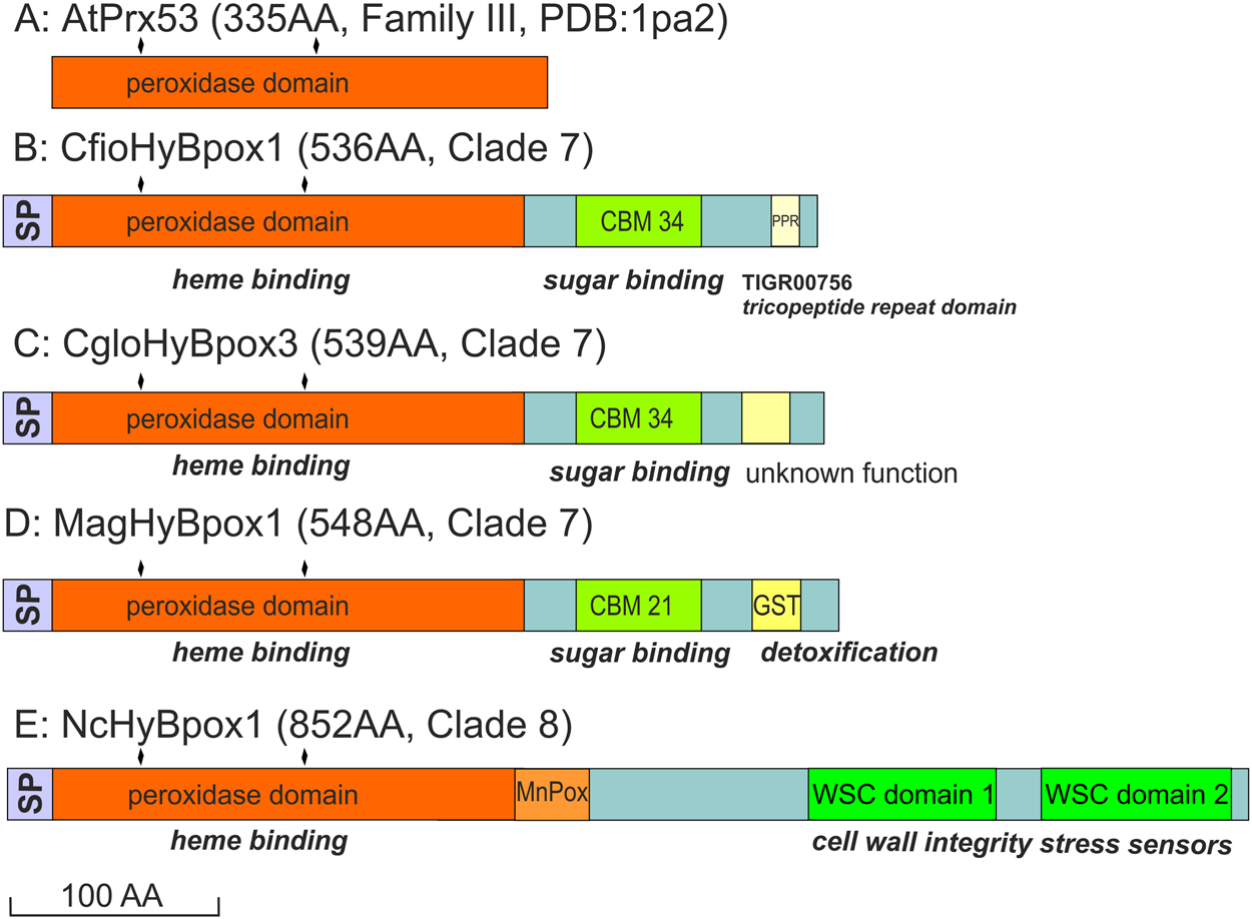
Schematic presentation of domain composition in selected Hybrid B heme peroxidase protein sequences. Four selected protein sequences of fungal Hybrid B peroxidases are compared with a sequence of a typical plant peroxidase. Abbreviations used: SP, signal peptide, MnPOX, motif known from a manganese peroxidase domain, CBM, carbohydrate binding module, WSC, cell wall stress-sensor component. Sequence motifs shown in yellow were identified with lower probability. Drawn to scale. Abbreviations of used peroxidase names are listed in Supplementary Table 1.

The monophyletic Hybrid B peroxidase subfamily with currently 114 full-ORF-length representatives can be subdivided in 9 main clades. Two of them are chytridiomycetous, three are basidiomycetous, and remaining four ascomycetous. In the well-resolved solely ascomycetous clade #7 formed by sequences from phytopathogenic fungi (detail presented in Fig. 2) we have discovered a unique fusion of a N-terminal heme peroxidase domain with two different C-terminal carbohydrate binding motifs (CBMs) that are presented schematically in Fig. 3. For the domain architecture analysis we have selected one typical sequence of a Hybrid B peroxidase from a hemibiotrophic pathogen *Magnaporthe oryzae* causing rice and wheat blast (i.e. MagHyBpox1) and two other sequences from related hemibiotrophs (i.e. CfioHyBpox1 and CgloHyBpox3 – abbreviations explained in Supplementary Table 1). Observed two domain composition is quite different from the longer Hybrid B variants in clade #8 containing - besides a conserved peroxidase domain - at least two similar WSC domains (Fig. 3, lower part) as described previously^14^. The WSC domain with the InterPro accession IPR002889 (or PF01822) was formerly described also as a putative carbohydrate binding domain. Mostly, it contains up to eight conserved cysteine residues that may be involved in several disulfide bridges. However, there is currently no evidence on its ability to specifically bind carbohydrates similar to above mentioned CBMs. A detailed functional analysis revealed that WSC proteins are typically highly O-glycosylated^22^ and that they can serve as cell wall integrity stress sensors^23^.

### Domain architecture of Hybrid B peroxidases

**Figure 4:**
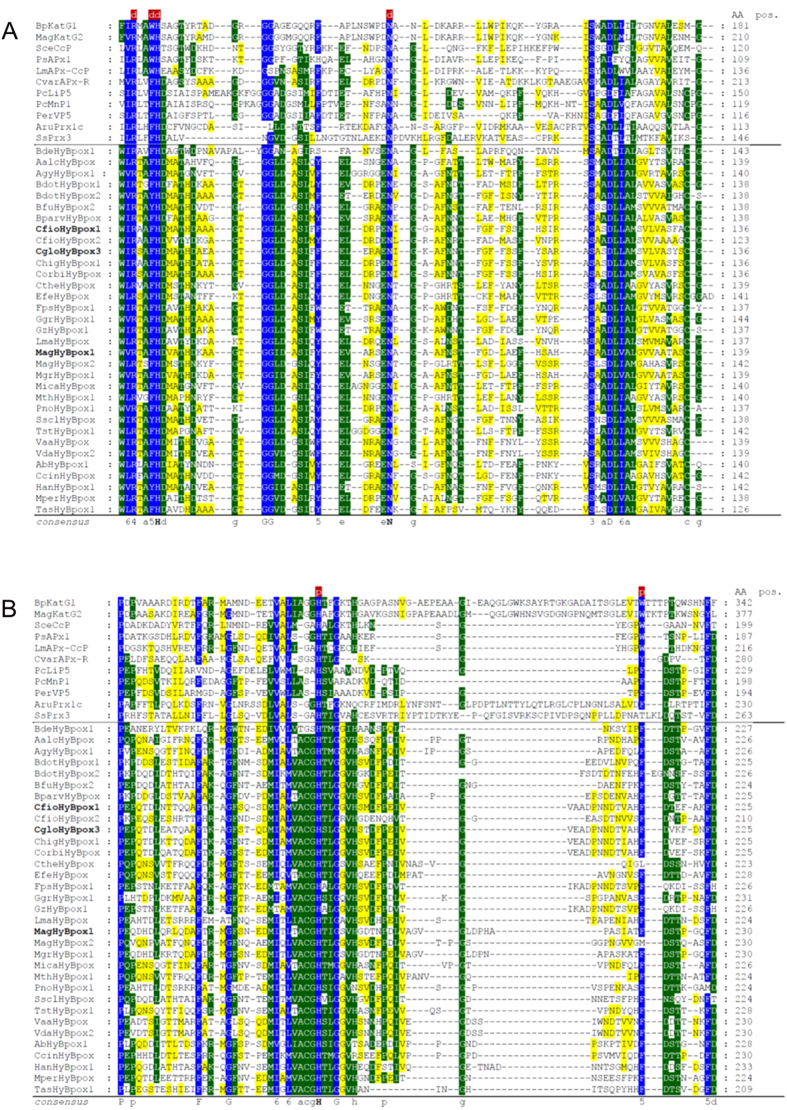
Multiple sequence alignment of 44 selected peroxidase-catalase superfamily members. Whole alignment in fasta format is deposited in Supplemental material 2. This part of the alignment shows highly conserved regions with essential residues of the heme peroxidase domain: (**A**) Region on the distal heme side and (**B**) region on the proximal side of the prosthetic heme group. Color scheme: blue > 90% conservation, green > 60% conservation, yellow > 25% conservation.

From the multiple sequence alignment (Fig. 4 and Supplementary material 2) it is obvious that the N-terminal peroxidase domains of Hybrid B peroxidases have the same length, mainly α-helical overall fold and highly conserved heme cavity as all other members of the peroxidase-catalase superfamily. It is expected that the prosthetic group is non-covalently bound in this typical pocket that was preserved during the long evolution of this superfamily. Important invariantly conserved catalytic residues include the distal Arg/His pair, which supports the deprotonation of H_2_O_2_ and the heterolytic cleavage of the peroxide bond. Described Arg/His pair is part of the conserved triad Arg108 – Trp111 – His112 (BpKatG1 numbering in the upper sequence of Fig. 4A). The third amino acid in the distal triad is involved in the formation of covalent adduct only in ancestral catalase-peroxidases (KatGs) but during the evolution it was substituted mainly with phenylalanine. The latter event is reflected by the conversion of bifunctional KatGs to monofunctional peroxidases^1^. Some rare and interesting variations within the whole superfamily are found only on the heme distal side in Hybrid B peroxidases, namely Arg69 – **Tyr72** – His73 (e.g. BdotHyBpox2 numbering) or even **Lys69** – **Tyr72** – His73 (SsclHyBpox numbering). The latter unique variant opens the question about the role of lysine in heterolytic peroxide bond cleavage. In any case, the distal histidine is apparently invariantly conserved in all known sequences of the whole superfamily. Besides this conserved triad an invariant Asn142 (BpKatG1 numbering) occurs at the distal side, which is involved in H-bonding and modulation of the p*K*_a_ of the above mentioned catalytic His^24^.

**Figure 5:**
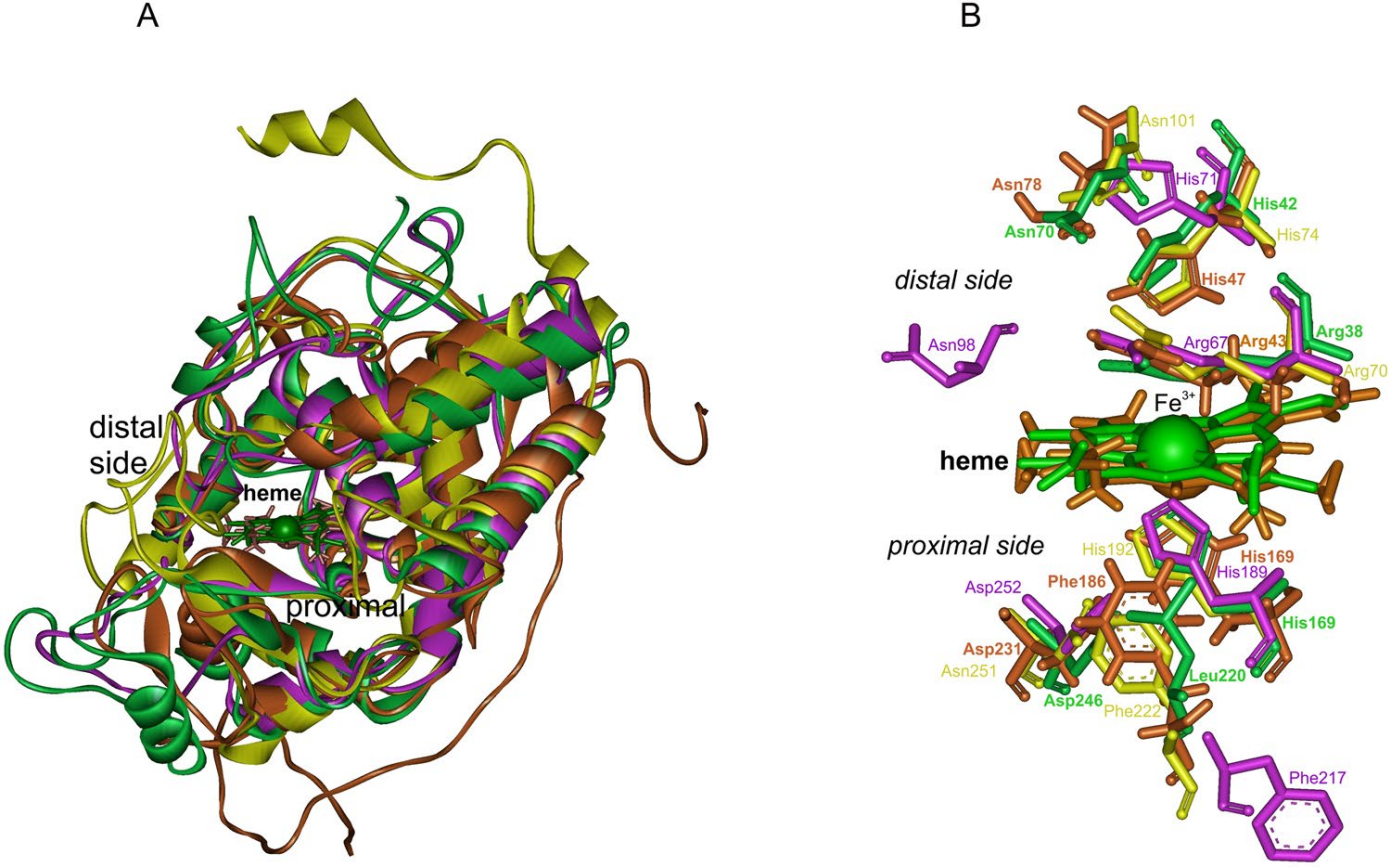
Structural comparison of various heme peroxidase domains from the peroxidase-catalase superfamily. (**A**) Experimentally determined 3D structures of a typical Family II fungal peroxidase (PDB code: 3FMU – a versatile peroxidase from *Pleurotus eryngii*) and a typical Family III plant peroxidase (PDB code: 1PA2 – *Arabidopsis thaliana* A2 peroxidase) are overlaid with two modelled structures of heme domains in MagHyBpox1 and CgloHyBpox3. Presented are the models with highest confidence values and alignment coverage obtained from Phyre 2 server. Four protein chains are displayed as solid ribbons. Color scheme: MagHyBpox1 yellow, CgloHyBpox3 violet, 3FMU brown and 1PA2 green. Also the prosthethic heme groups of two experimentally determined structures are presented as colored sticks in the superimposed active centers (the same color as the respective protein chain of 3FMU & 1PA2). (**B**) The same structural overlap but with hidden polypeptide chains giving emphasis on the conserved essential residues on the distal and proximal side of the prosthetic heme group. Color scheme for the proteins is the same as in (**A**).

At the proximal side the heme ligand His279 (Fig. 4B) and its H-bonding partner Asp389 (BpKatG1 numbering, see Supplementary material 2) are fully conserved in the whole superfamily and contribute to the stabilization of the ferric resting state. Together with a Trp or Phe they constitute the proximal triad. In almost all Hybrid B peroxidases a Phe is found (Phe216, BdotHyBpox2 numbering) whereas in Family I peroxidases a Trp is located at this position. Figure 5 demonstrates the high level of structural conservation within this superfamily by comparing the crystal structures of a fungal and plant peroxidase as well as two Phyre-predicted structural models of Hybrid B-peroxidases.

Almost all members of this superfamily are one domain proteins consisting of the peroxidase domain only. Exceptions are catalase-peroxidases and Hybrid B peroxidases (and few Hybrid A members). At the basis of evolution of the whole superfamily two-domain bifunctional catalase-peroxidases are found^25^. They have a N-terminal catalytic heme domain and a shorter gen-duplicated homologous (heme-free) domain that supports the maintenance of the overall and heme cavity architecture^26^. The phylogenetic origin of the C-terminal domain is still under discussion^15^. Anyway, in course of further evolution this C-terminal domain was lost. There are rare exceptions detected only among Hybrid A peroxidases where the second domain still remained preserved^27^. However, the vast majority of members of the various subfamilies (except Hybrid B peroxidases) consist of only a single heme peroxidase domain (overview in PeroxiBase^10^ http://peroxibase.toulouse.inra.fr/).

### Structural analysis of newly discovered CBMs present in phytopathogenic Ascomycetous Hybrid B heme peroxidases

**Figure 6:**
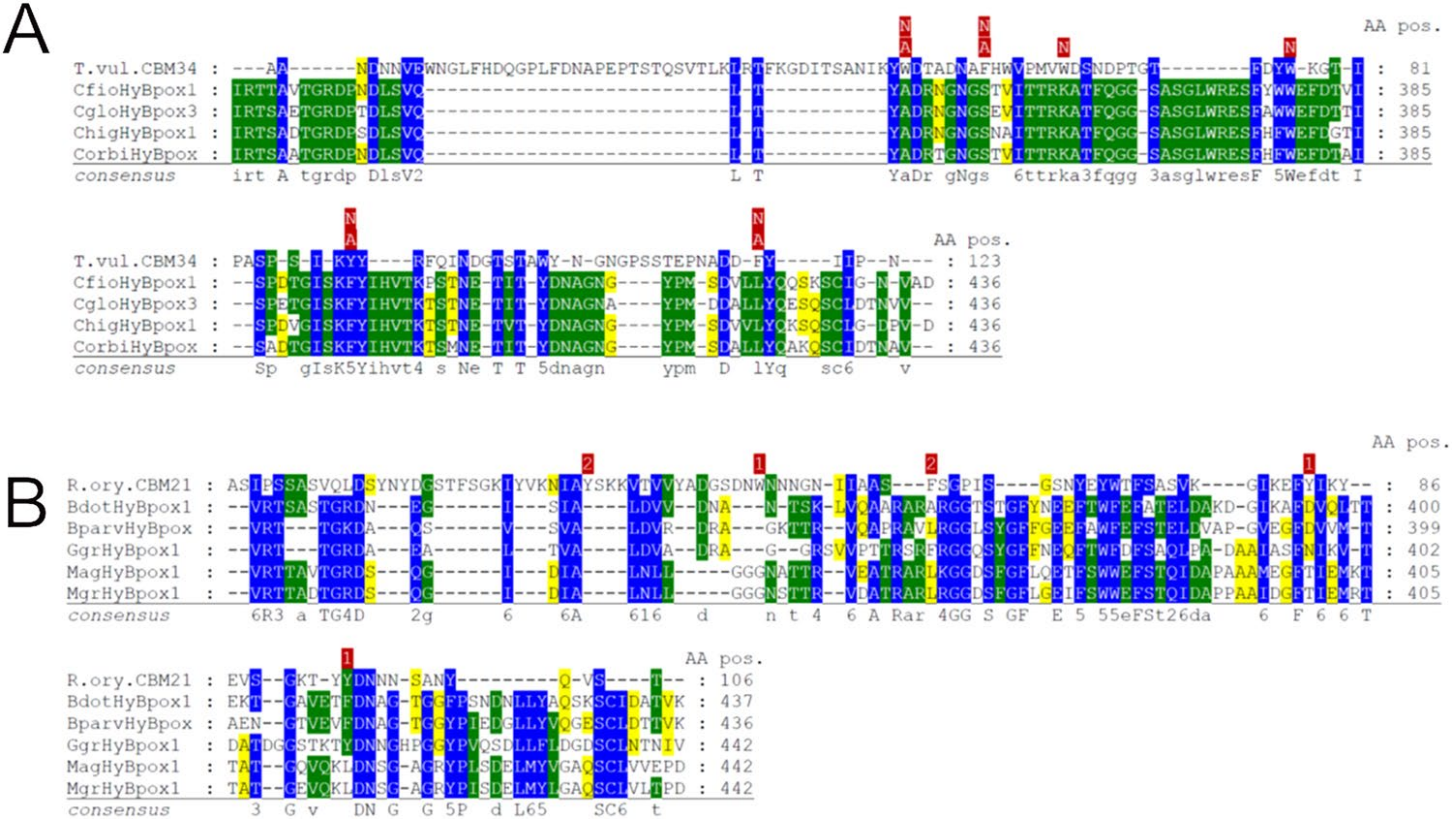
Multiple sequence alignment of selected ascomycetous Hybrid B peroxidases with starch-binding domains of two distinct CBM families. (**A**) CBM 34 motif with 3 closely related HyBpox representatives (**B**) CBM 21 motif with 4 closely related hyBpox representatives. Color scheme: blue > 90% conservation, green > 60% conservation, yellow > 25% conservation.

All Hybrid heme B peroxidases are fused proteins consisting of the highly conserved heme peroxidase domain and at least one non-homologous and non-catalytic C-terminal domain (Figs 3 and 6). The C-terminal fusions are comprised of either multiple WSC domains (clade #8 in Figs 2 and 3) or a single carbohydrate binding motif with additional short variable motifs with mostly unknown function (clade #7). In contrast to our preliminary analysis of the C-terminal domains^14^ it is now obvious that not all Hybrid B peroxidases contain a WSC domain and the variability in this region of the fused peroxidases is much higher than expected before.

**Figure 7:**
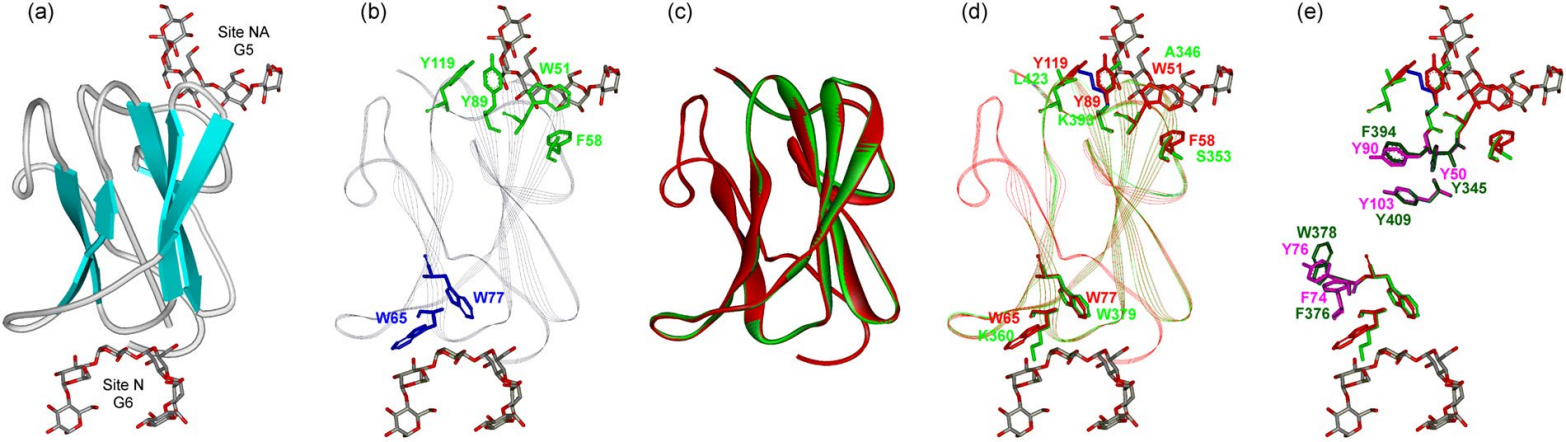
Structural comparison of second domain of ascomycetous Hybrid B peroxidases with starch-binding domain of the family CBM34. (**a**) Structure of the real family CBM34 starch-binding domain from the family GH13 α-amylase TVA-I from *Thermoactinomyces vulgaris* (PDB code: 1UH4^38^) with two binding sites, the site N and the site NA with bound maltohexaose (G6) and maltopentaose (G5), respectively. (**b**) Aromatic residues responsible for saccharide binding acting in the sites N (blue) and NA (green). (**c**) Superimposed real CBM34 from *T. vulgaris* TVA-I (red) with CBM34 models from fungal Hybrid B heme peroxidases - CfioHyBpox1 (blue) and CgloHyBpox3 (green) - covering 87 C_α_-atoms with a 0.15 Å root-mean square deviation. (**d**) Binding residues in the two carbohydrate binding sites in the real CBM34 structure (red) and their counterparts in both fungal peroxidases CBM34-like models (green and blue); only the CgloHyBpox3 residues being labelled. (**e**) Emphasis on aromatic residues from the real CBM34 (magenta) that, although not involved in carbohydrate binding, have their aromatic counterparts in both CBM34-like structures from fungal Hybrid B heme peroxidases (cyan and dark green); only the CgloHyBpox3 residues being labelled.

**Figure 8:**
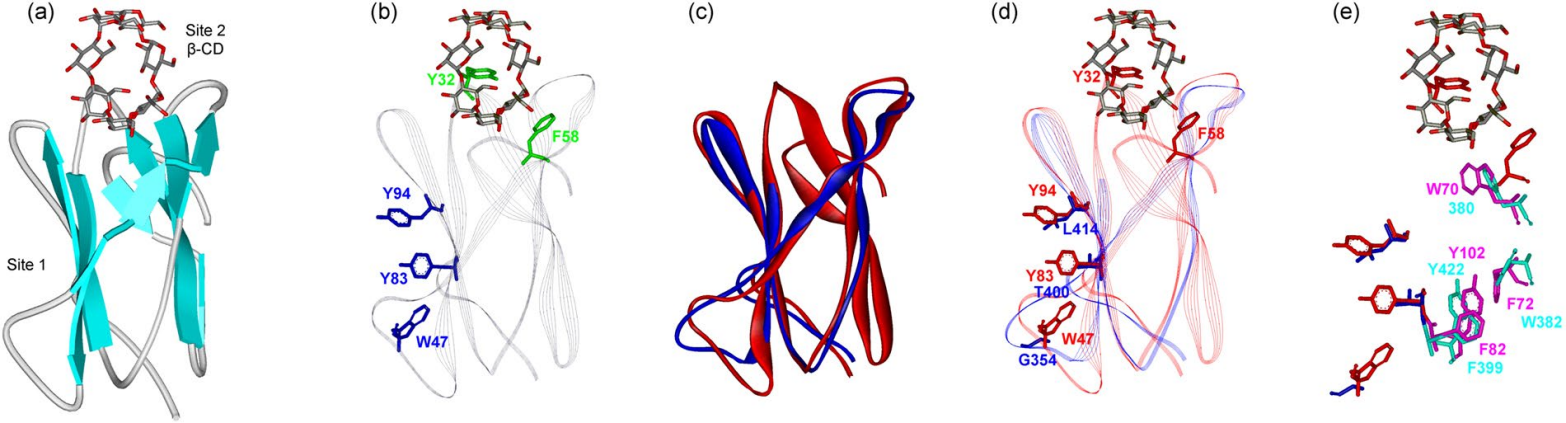
Structural comparison of second domain of ascomycetous Hybrid B peroxidases with starch-binding domain of the family CBM21. (**a**) Structure of the real family CBM21 starch-binding domain from the family GH15 glucoamylase from *Rhizopus oryzae* (PDB code: 2V8L^40^) with two binding sites, the site 1 (with no saccharide bound) and the site 2 complexed with β-cyclodextrin (β-CD). (**b**) Aromatic residues responsible for saccharide binding acting in the sites 1 (blue) and 2 (green). (**c**) Superimposed real CBM21 from *R. oryzae* glucoamylase (red) with CBM21 model from fungal Hybrid B heme peroxidase MagHyBpox1 (blue) covering 64 C_α_-atoms with a 1.17 Å root-mean square deviation. (**d**) Binding residues in the two carbohydrate binding sites in the real CBM21 structure (red) and their counterparts in the site 1 of the fungal peroxidase CBM21-like model (blue); in the site 2, there were no corresponding residues in the overlap. (**e**) Emphasis on aromatic residues from the real CBM21 (magenta) that, although not involved in carbohydrate binding, have their aromatic counterparts in the CBM21-like structure from the fungal Hybrid B heme peroxidase (cyan).

Our structural analysis (Figs 7 and 8) clearly demonstrates that these CBMs present in phytopathogenic Ascomycetous peroxidases belong to CBM21 and CBM34 families. We have selected CfioHyBpox1, CgloHyBpox3 and MagHyBpox1 that revealed in the first round of screening the highest probability for the presence of CBM domains by using the CDvist suite^28^. It has to be emphasized that both CBM21 and CBM34 belong to the so-called raw starch-binding domains (SBD) found typically as modules of various microbial amylolytic enzymes^29^. Among 81 currently known CBM families there are at least 13 verified raw starch-binding domains as classified in the CAZy database (http://www.cazy.org/ ^30^) and, indeed, some of them have already been identified in non-amylolytic enzymes^29^. For example, CBM20 was found in the mammalian genethonin-1^31^ and laforin^32^ as well as in fungal lytic polysaccharide monooxygenases^33^, whereas CBM48 was detected in the plant SEX4 protein^32^ and the β-subunit of AMP-activated protein kinase^34^.

With regard to Hybrid B heme peroxidases the eventual presence of any CBM with assumed raw starch-binding capability is highly interesting. Despite the fact that MagHyBpox1 may contain CBM21, while both CfioHyBpox1 and CgloHyBpox3 possess CBM34, structural superimposition of their models with experimentally verified CBM21 and CBM34 templates clearly demonstrate that the overall respective folds, i.e. a typical immunoglobulin-like fold (β-sandwich) consisting of several antiparallel β-strands, have been preserved (Figs 7C and 8C). Note, that the CBM models of MagHyBpox1, CfioHyBpox1 and CgloHyBpox3 were produced allowing the Phyre-2 server to choose the best templates, which were CBM21 from *Rhizopus oryzae* glucoamylase (PDB code: 2DJM^35^) for MagHyBpox1 (residues Asp345-Asp426) and CBM34 from *Thermoactinomyces vulgaris* α-amylase TVA-I (PDB code: 1JI1^36^) for both CfioHyBpox1 (residues Ile326-Gln426) and CgloHyBpox3 (residues Ile326-Glu426). In each case the models were selected in an effort to take into account the most appropriate combination of the three parameters confidence, sequence identity and alignment coverage.

Once the overall fold of CBM21 and CBM34 analogs in these phytopathogenic Ascomycetous peroxidases has been recognized, it was relevant to find out whether also the residues responsible for carbohydrate binding in the two CBMs have been conserved in peroxidases. In general, there is at least one, but usually two starch-binding sites in a CBM known as a starch-binding domain^29, 37^. This is also the case of CBM34 (Fig. 7A,B) and CBM21 (Fig. 8A,B). Saccharide binding is provided mostly by aromatic residues involved in stacking interactions, but hydrogen bonds may also be involved^29, 36–40^. Although no saccharide was seen complexed at binding site 1 (Fig. 8A,B) in the three-dimensional structure of CBM21 from the *Rhizopus oryzae* glucoamylase (PDB codes: 2DJM, 2V8L), the relevant aromatic residues are present at both binding sites^40^. Comparison of saccharide binding residues from known CBM21s and CBM34s with putative CBMs from Hybrid B peroxidases (Figs 7D and 8D) shows that out of the six aromatic residues of characterized CBM34s (Fig. 7B), only Trp77 has the corresponding aromatic residue in the respective CBM34 models from CfioHyBpox1 (i.e. Trp379) and CgloHyBpox3 (Fig. 7D). The situation in putative CBM21 from MagHyBpox1 is even less convincing, i.e. out of the five aromatic residues of the two binding sites of characterized CBM21 (Fig. 8B), no corresponding aromatic amino acid was found. Moreover, the second binding site could not be identified due to incompleteness of model (Fig. 8C,D).

However, there are several other aromatic residues, although temporarily with no assigned functional role, positioned “inside” the CBM, which are found in real amylolytic starch-binding domains and the Hybrid B peroxidase models (Figs 7E and 8E). Five such residues can be seen in CBM34 of both CfioHyBpox1 and CgloHyBpox3 (Fig. 7E) and four in CBM21 of MagHyBpox1 (Fig. 8E). A similar observation has been reported for other starch-binding domains from the family CBM41^29, 41^, for which it has been hypothesised that these aromatic positions (neither totally conserved, nor functional role ascribed based on solved structures) may represent a relict from a primordial CBM ancestor before the current CBMs specialized during evolution.

A functional connection of a heme peroxidase with carbohydrate binding motifs thus observed among Hybrid B peroxidases from various important hemibiotrophic Ascomycetes might have significant impact for their phytopathogenicity. Transcripts of corresponding genes are currently detected in fungal families Magnaporthaceae & Glomerellaceae within mRNA libraries either non-induced or induced with some kind of oxidative stress (e.g. GenBank-EST database accession numbers JZ969979.1, JZ970399.1 or DR621480.1). The physiologically observed oxidative burst accomplished by a prompt accumulation of reactive oxygen species mainly from the action of plant host NADPH oxidases represents the main streamline of the apoplastic immunity^42^. A concerted action of peroxide bond cleavage with a specific binding on integral cell-wall carbohydrates can counteract the plant defence pathways and allow the fungal pathogen to survive within the host tissue. Concerning the taxonomy spectrum of organisms found currently in the families CBM21 or CBM34, the former can be considered a eukaryotic family with a majority of various amylases of yeast and fungal origin, whereas the latter is yet a solely prokaryotic family with an unambiguous dominance of bacterial amylolytic enzymes^30^. To identify a homologue of CBM21, which is a typical fungal domain, among fungal Hybrid B peroxidases may thus not be so surprising, but to reveal a homologue of a typically bacterial CBM34 in a fungal hybrid B peroxidase should be of interest. Moreover, both CBM21 and CBM34 are best known as non-catalytic modules of amylolytic enzymes, which help their catalytic domains to bind and degrade raw starch or, in a wider sense, the α-glucans related to and/or derived from starch^29, 35–41^. Since, however, the residues responsible for binding the α-glucans in both CBM21 and CBM34 have not been found to be conserved in their counterparts from Hybrid B peroxidases, it is possible to expect also some changes in target bound carbohydrates, even in terms of their stereochemistry, i.e. a change to β-glucans. To determine the exact role these CBMs may play in the function of Hybrid B peroxidases represents therefore a relevant challenge for experiments on purified proteins that are already being undertaken.

## Conclusion

The phylogenetic reconstruction of the peroxidase-catalase superfamily reveals three well resolved families and two distantly related polyphyletic Hybrid A (ascorbate-cytochrome *c* peroxidases) and monophyletic Hybrid B enzymes. The latter are unique fusion proteins containing a N-terminal highly conserved peroxidase domain and C-terminal domain comprised of variable carbohydrate binding motifs of two different types. The here observed unique domain fusion between a heme peroxidase and a CBM domain can open new horizons of future research exploring the physiological impact of the oligosaccharide binding domain(s) on the peroxidase function which might include hydrogen peroxide degradation during oxidative burst and/or site specific plant polymer degradation reactions in biotrophic and hemibiotrophic fungal pathogens.

## Materials and Methods

### Sequence data collection and multiple sequence alignment

All sequence data used for this analysis were collected from public databases. Protein sequences of herein analysed peroxidases were mainly from PeroxiBase^10^ at http://peroxibase.toulouse.inra.fr. Only in the case that a particular peroxidase sequence was not (yet) available in PeroxiBase corresponding Uniprot accession was used. All analysed peroxidase sequences are representative for the whole peroxidase-catalase superfamily divided in three main families and twelve subfamilies currently counting almost 8,800 manually annotated & curated sequences in PeroxiBase (in total already over 23,300 hits, provided mostly as automatic genomic annotation in InterPro database). Multiple sequence alignment of 500 selected full length protein sequences was performed with Muscle program^43^ implemented in MEGA 7 package. Optimized alignment parameters were: gap open −0.8 gap extend −0.05, hydrophobicity multiplier 0.9. Maximum of performed alignment iterations was set to 1,000. The used clustering method was UPGMB, for other interactions NJ and minimal diagonal length was set to 28. Alignment was inspected mainly for the presence of seven conserved catalytic residues on both distal and proximal sides involved in catalysis and binding of the heme prosthetic group^19, 44^ and further refined in GeneDoc^45^. Ambiguously aligned regions were excluded from further analysis. After inspection and refinements the final alignment used for molecular phylogeny contained 500 full length sequences from all subfamilies of the peroxidase-catalase superfamily. For bifunctional catalase-peroxidases analysed thoroughly in previous studies^12, 14^ only the sequences of N-terminal domain known to bind the prosthetic heme group were used and not their gene-duplicated C-terminal (heme-free) counterpart.

### Molecular phylogeny reconstruction

Molecular phylogeny was first reconstructed using the MEGA package, version 7^46^. Muscle-aligned protein sequences including all sequences with currently known 3D structures were subjected to Maximum-Likelihood (ML) method of this package. Following optimised parameters were applied: 100 bootstraps, WAG model^20^ of amino acid substitutions with four discrete gamma categories. The branch swap filter was set to very strong and the number of threads was set to 1. The branching patterns for particular subfamilies were presented with the Tree Explorer program of the MEGA 7 package in the rectangular form. The same protein alignment of 500 peroxidase sequences was then subjected to phylogenetic reconstruction using MrBayes 3.2.6 suite^47^. For calculating substitution rates the WAG model^20^ applying invariant gamma option was used with 4 discrete gamma categories. For diagnostics a relative burn-in of 25.0% was applied. Majority consensus tree was obtained from all credible topologies sampled by MrBayes over 3,000,000 generations with finally achieved standard deviation of split frequencies below 0.09 (recommended limit 0.10). Resulting trees were displayed and annotated with Interactive tree of life (iTOL v.3^48^) in a circular form with transformed branches.

### Identification of introns and exons and prediction of signal sequences

Search for donor & acceptor splice sites in (mostly) putative fungal hybrid peroxidase genes was performed. For this purpose the program suite NetAspGene 1.0 of the CBS server was used (http://www.cbs.dtu.dk/services/NetAspGene/ ^49^). GT-AG consensus sequence for the borders between exons and introns was present in most but not all hybrid peroxidase genes. Detailed output for each particular gene is presented in PeroxiBase^10^.

Putative signal sequences for protein secretion were revealed using the predictive algorithm of the program SignalP 4.1 (http://www.cbs.dtu.dk/services/SignalP/ ^50^). The appropriate prediction database was chosen according to determined phylogenetic relationship of the analysed sequence. Those sequences that were found as intracellular with this approach were further subjected to subcellular localization analysis using TargetP 1.1 from the same online suite^50^.

### Analysis of domain assembly, sequences and tertiary structures of carbohydrate binding motifs (CBMs)

CDvist^28^ was used as a comprehensive visualization tool to delineate the presence of distinct domains in various fused proteins of the peroxidase-catalase superfamily. Following optimized parameters were used for screening: TMHMM for transmembrane prediction, HMMER3, domain split up to 5.0%, HH search 1 Pfam 75.0% cutoff, gap length 50aa, HH search 2 CDD 75.0% cutoff, gap length 50aa, HH search 3 PDB 75.0% cutoff, gap length 30aa, HH search 4 SCOP 75.0% cutoff, gap length 30aa, HH search 5 TIGR 75.0% cutoff, gap length 50aa and HHblits Uniprot with probability cutoff 60.0.

All used CBM sequences were retrieved from the UniProt knowledge database^51^; http://www.uniprot.org/) and/or GenBank^52^; http://www.ncbi.nlm.nih.gov/genbank/). The partial alignment, covering only the predicted CBM domain, was performed using the program Clustal-Omega available at the European Bioinformatics Institute’s web-site (http://www.ebi.ac.uk/). In order to maximize similarities, the alignment was manually tuned taking into account previous bioinformatics studies^37, 53–55^.

Three-dimensional structures were retrieved from the Protein Data Bank (PDB^56^; http://www.rcsb.org/pdb/) for representatives of the individual CBM families, i.e.: CBM21 (PDB code: 2V8L^40^) and CBM34 (PDB code: 1UH4^38^). Three-dimensional models for domains without experimental 3D structure were created with the Phyre-2 server^57^ (http://www.sbg.bio.ic.ac.uk/phyre2/) employing the “Normal” modelling mode. Obtained structures were superimposed using the program MultiProt^58^ (http://bioinfo3d.cs.tau.ac.il/MultiProt/) and displayed with the WebLab Viewer Lite programme (Accelrys Inc.).

### Accession codes

Of all peroxidases used in this work can be retrieved in PeroxiBase^10^ (http://peroxibase.toulouse.inra.fr/) and are listed in Supplementary Table 1.

## Electronic supplementary material

Table with complete sequence list

Supplementary Material
